# Supplementary material for: A short-term memory trace persists for days in the mouse hippocampus
Source: Commun Biol. 2022 Nov 3;5:1168. doi: 10.1038/s42003-022-04167-1 (PMC9633825; doi:10.1038/s42003-022-04167-1)
Supplement: Supplementary file 2 — Supplementary Information [file 42003_2022_4167_MOESM2_ESM.pdf]

SUPPLEMENTARY INFORMATION

A short-term memory trace persists for days in the mouse hippocampus

Authors

Maha E. Wally[1,2,3,4], Masanori Nomoto[1,2,3], Kareem Abdou[1,2,3,5], Emi Murayama[1,2,3], Kaoru Inokuchi[1,2,3*]

Affiliations

[1]Research Center for Idling Brain Science, University of Toyama, Toyama 930-0194, Japan

[2]Department of Biochemistry, Graduate School of Medicine and Pharmaceutical Sciences, University of Toyama, Toyama 930-0194, Japan

[3]CREST, JST, University of Toyama, Toyama 930-0194, Japan

[4]Pharmacology Department, Faculty of Pharmacy, The British University in Egypt, Cairo 11837, Egypt

[5]Department of Biochemistry, Faculty of Pharmacy, Cairo University, Cairo 11562, Egypt

[*]Correspondence: inokuchi@med.u-toyama.ac.jp

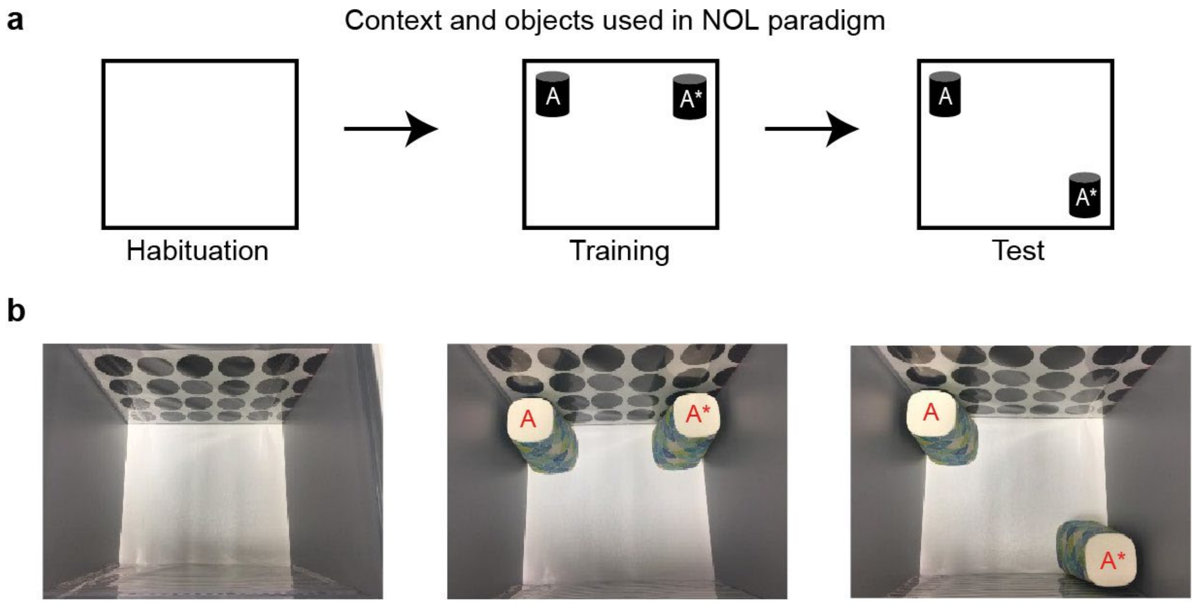

Supplementary Figure 1. Novel object location paradigm layout. a) Context and objects layout; object A: remains in its location during test, object A*: changes its location during test. Object location was counterbalanced across mice. **b)** Photos of context and objects used, 2 walls of the context were covered with spatial cues to aid the mice in navigation, one wall had circular patterns and the opposite wall had stripes pattern. (see the Methods section for a full description of the NOL paradigm).

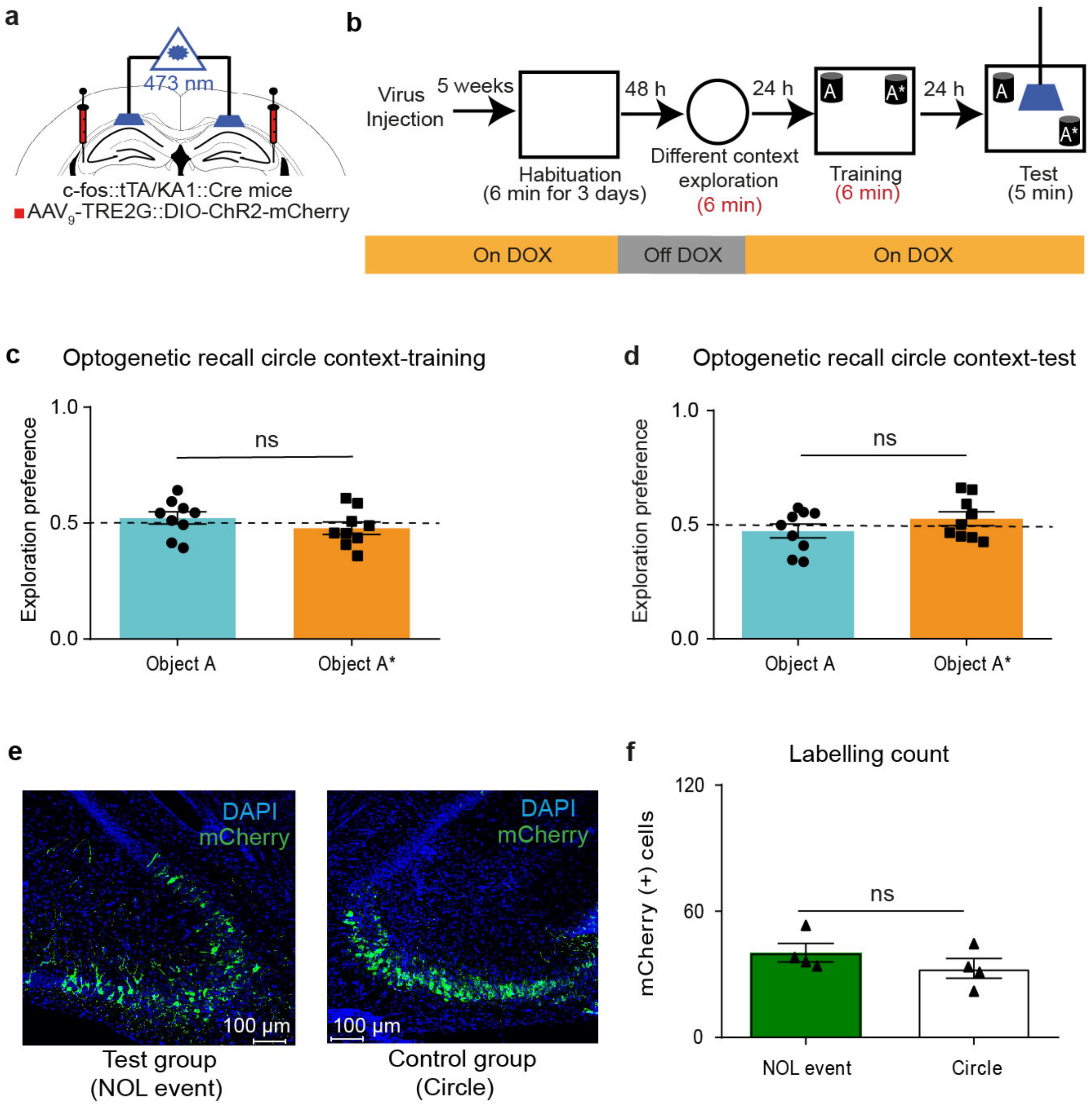

Supplementary Figure 2. Optogenetic activation of a non-NOL engram does not induce artificial recall of the NOL-STM engram. a) Diagram of AAV injection into CA3, guide cannula and optic fiber placement into CA1. **b)** Experimental design for labelling and optogenetic recall of a non-NOL STM engram (circle context). **c-d)** Exploration preference for each object during NOL training (b) and NOL test (c) sessions (n = 9). **e)** Representative section of CA3 showing mCherry protein expression for test group with labelled NOL event and control group with labelled circle (scale bar = 100 µm, DAPI: 4′,6-diamidino-2-phenylindole). **f)** Number of mCherry-positive cells in test group with labelled NOL event and control group with labelled circle (n=4 mice, 3 replicates each). Comparisons were

made using paired student's t-test for comparing object A and object A* in the same group (b-c) and unpaired student's t-test (e); ns, not significant ($P > 0.05$). Data are presented as the mean ± SEM.

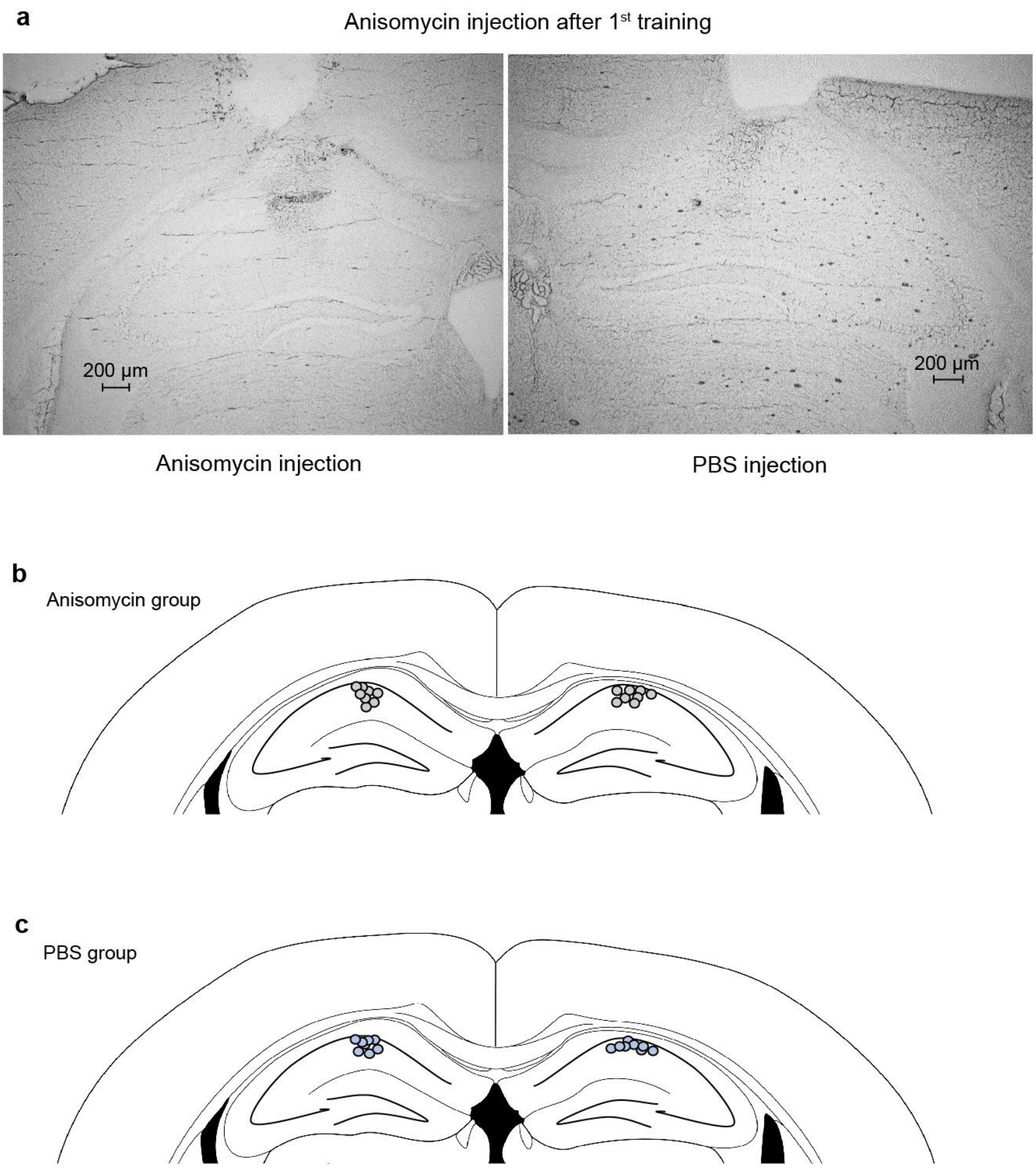

Supplementary Figure 3. Cannula tip placement in mice infused with anisomycin and PBS after the first training. a) Representative image of anisomycin injection (left) and PBS injection (right), scale bar = 200 µm. **b)** Cartoon showing injection traces of anisomycin group (n = 8). **c)** Cartoon showing injection traces of PBS group (n = 8).

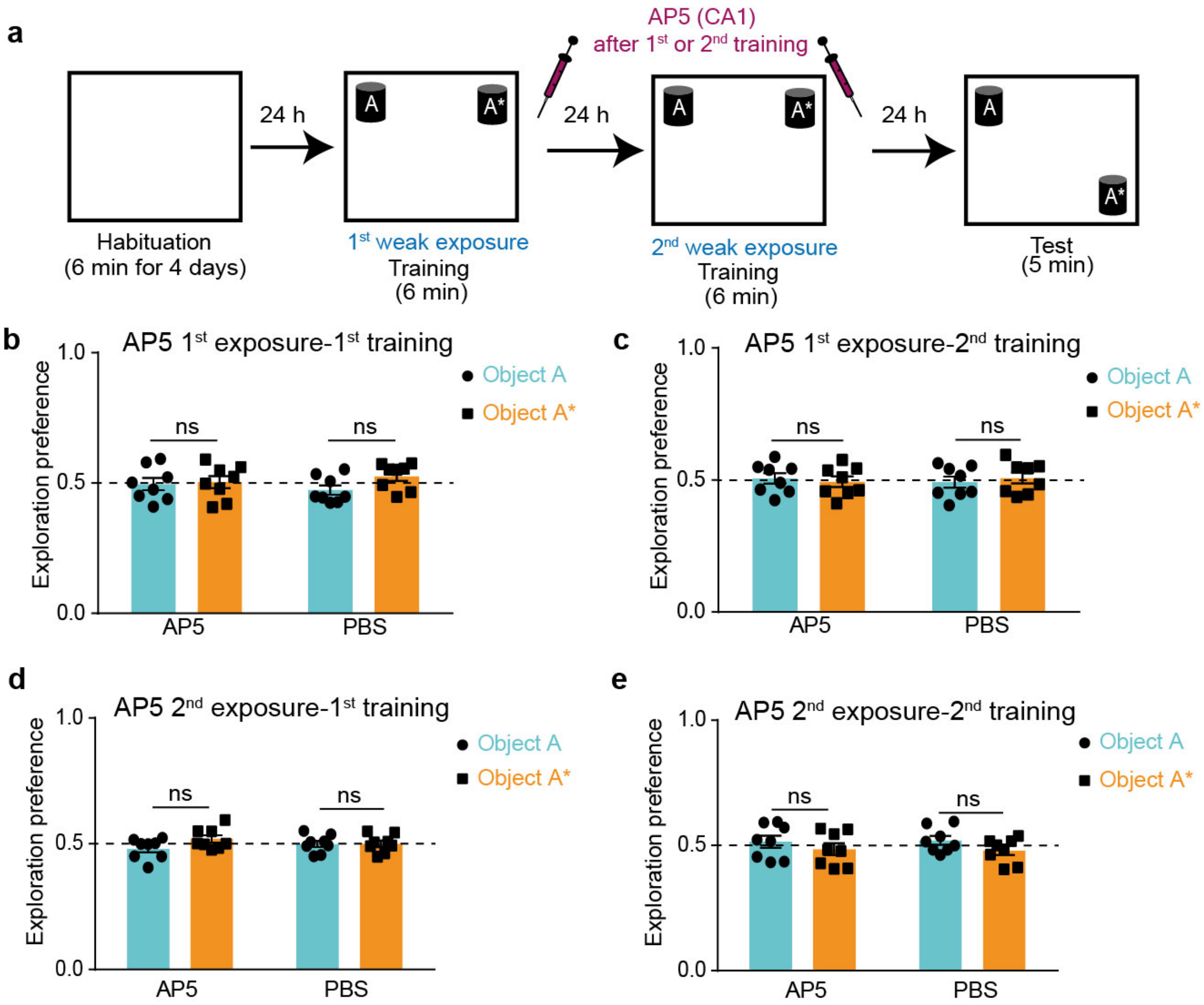

Supplementary Figure 4. Training sessions for N-methyl-D-aspartate receptor blockade experiments. a) NOL two-training paradigm with 1-day interval in between with AP5 injection after either the first or the second training sessions into the CA1 region. **b-c)** Exploration preference for each object during the first (B) or the second (C) training sessions for AP5- (n = 8) and PBS- (n = 8) injected groups after the first training session. **d-e)** Exploration preference for each object during the first (D) or the second (E) training sessions for AP5- (n = 8) and PBS- (n = 8) injected groups after the second training session. Comparisons were made using paired student's t-test for comparing object A and object A* in the same group; ns, not significant ($P > 0.05$). Data are presented as the mean ± SEM.

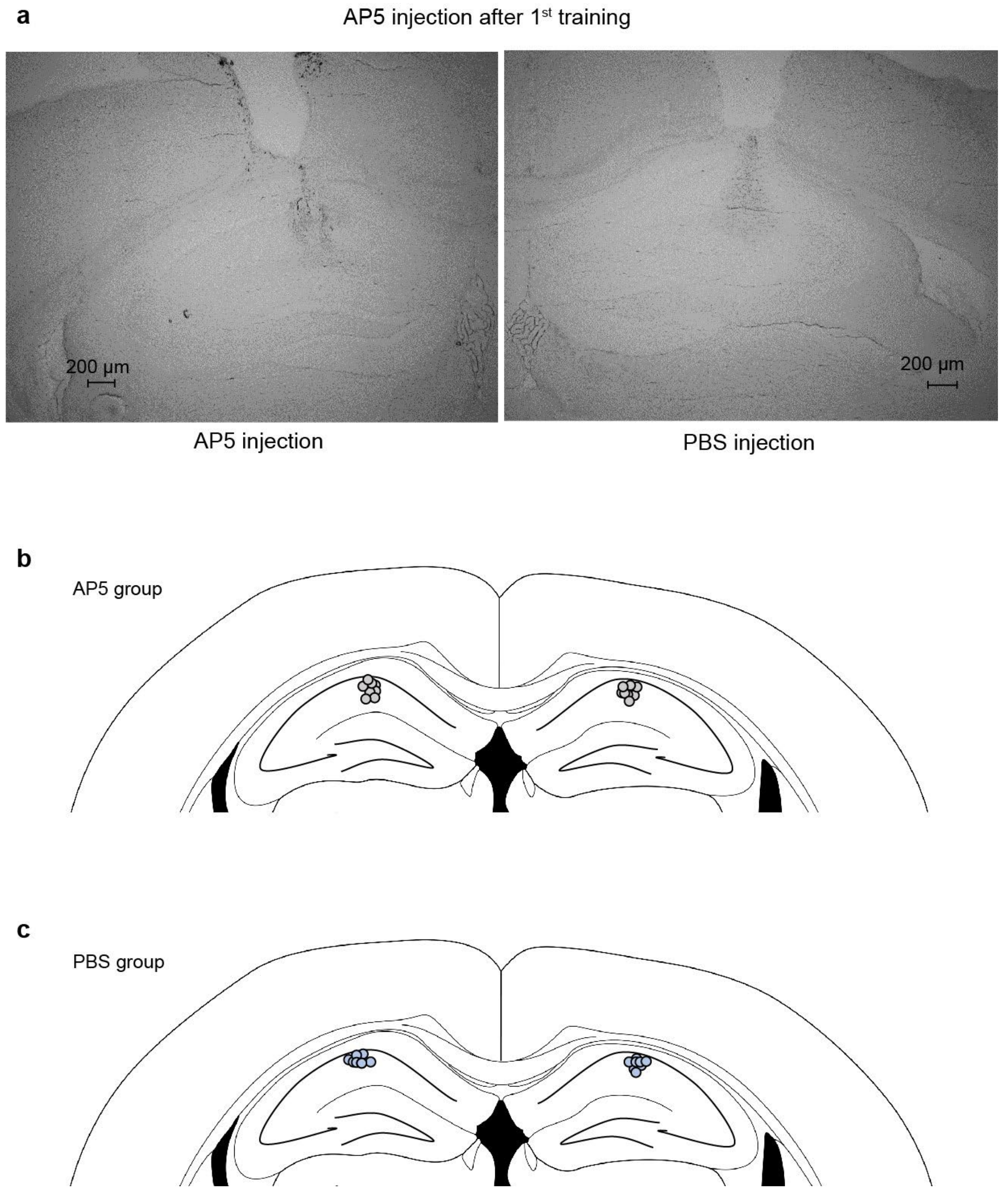

Supplementary Figure 5. Cannula tip placement in mice infused with AP5 and PBS after the first training. a) Representative image of AP5 injection (left) and PBS injection (right), scale bar = 200 μm. **b)** Cartoon showing injection traces of AP5 group (n = 8). **c)** Cartoon showing injection traces of PBS group (n = 8).

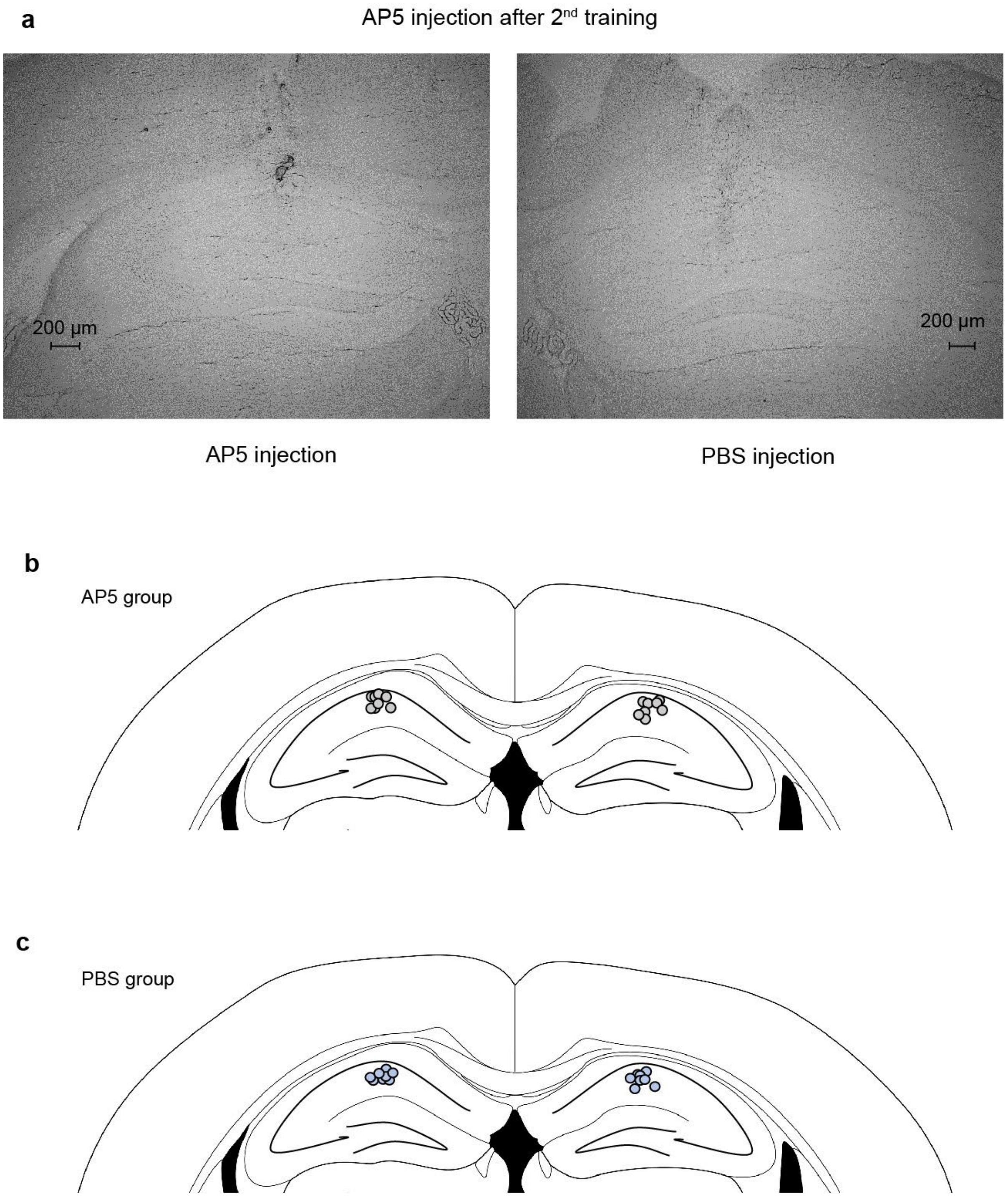

Supplementary Figure 6. Cannula tip placement in mice infused with AP5 and PBS after the second training. a) Representative image of AP5 injection (left) and PBS injection (right), scale bar = 200 μm. **b)** Cartoon showing injection traces of AP5 group (n = 8). **c)** Cartoon showing injection traces of PBS group (n = 8).

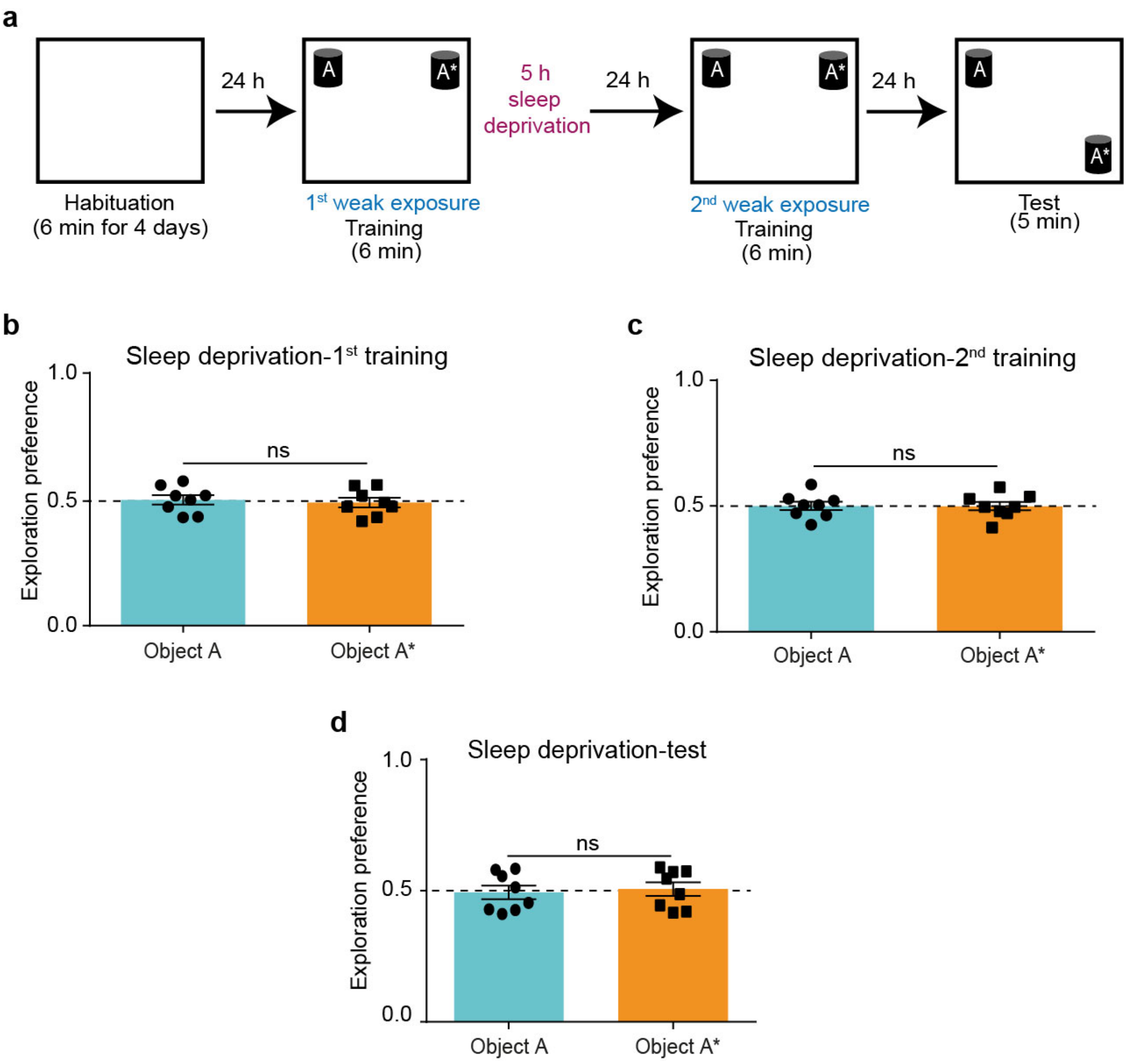

Supplementary Figure 7. Post-learning sleep is required to preserve STM trace. a) NOL two-training paradigm with 1-day interval with 5-hour sleep deprivation after the first training session. **b-c)** Exploration preference for each object during the first (B) and the second (C) training sessions (n = 8). **d)** Exploration preference for each object during the test session (n = 8). Comparisons were made using paired student's t-test for comparing object A and object A* in the same group; ns, not significant ($P > 0.05$). Data are presented as the mean ± SEM.

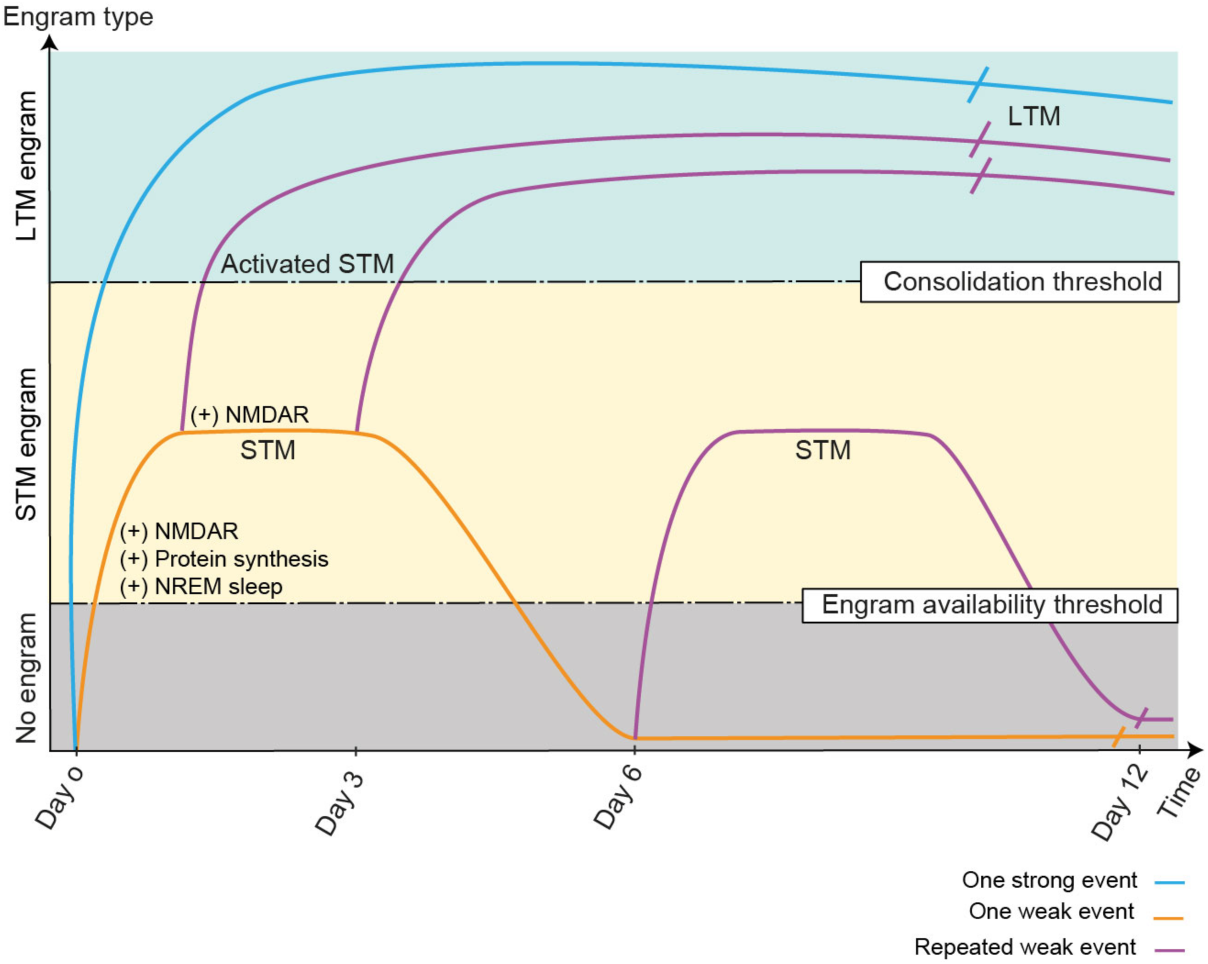

Supplementary Figure 8. Hypothesized model for STM vs. LTM engrams. One strong event is sufficient to consolidate an LTM, which is stored in the form of an active engram that can be naturally recalled. One weak event is not sufficient for consolidation; however, it forms an STM stored in the form of an engram and this storage requires post-learning NMDAR activation, new protein synthesis, and NREM sleep. A repeated weak event can activate this STM engram by consolidation within its lifetime (<6 days) and this activation process requires post-learning NMDAR activation as well. However, if the second weak event is repeated after 6 days, the first STM engram is no longer available and the repeated weak event is then processed as a new event by forming a new STM engram with similar properties. This model does not take into account the initial period of transient same-day STM recall, but rather focuses on the detailed long-term storage of STM for the sake of clarity.
