# Supplementary material for: A short-term memory trace persists for days in the mouse hippocampus
Source: Commun Biol. 2022 Nov 3;5:1168. doi: 10.1038/s42003-022-04167-1 (PMC9633825; doi:10.1038/s42003-022-04167-1)
Supplement: Supplementary file 3 — Description of Additional Supplementary Files [file 42003_2022_4167_MOESM3_ESM.pdf]

Description of Additional Supplementary Files

File name: Supplementary Data 1
Description: Sampling and statistical analysis details.

File name: Supplementary Data 2
Description: Source Data file.
