# Supplementary material for: A short-term memory trace persists for days in the mouse hippocampus
Source: Commun Biol. 2022 Nov 3;5:1168. doi: 10.1038/s42003-022-04167-1 (PMC9633825; doi:10.1038/s42003-022-04167-1)
Supplement: Supplementary file 6 — Reporting Summary [file 42003_2022_4167_MOESM6_ESM.pdf]

nature portfolio

Corresponding author(s): Kaoru Inokuchi

Last updated by author(s): Oct 19, 2022

Reporting Summary

Nature Portfolio wishes to improve the reproducibility of the work that we publish. This form provides structure for consistency and transparency in reporting. For further information on Nature Portfolio policies, see our Editorial Policies and the Editorial Policy Checklist.

Statistics

For all statistical analyses, confirm that the following items are present in the figure legend, table legend, main text, or Methods section.

n/a	Confirmed	
☐	☒	The exact sample size (*n*) for each experimental group/condition, given as a discrete number and unit of measurement
☐	☒	A statement on whether measurements were taken from distinct samples or whether the same sample was measured repeatedly
☐	☒	The statistical test(s) used AND whether they are one- or two-sided<br>*Only common tests should be described solely by name; describe more complex techniques in the Methods section.*
☐	☒	A description of all covariates tested
☐	☒	A description of any assumptions or corrections, such as tests of normality and adjustment for multiple comparisons
☐	☒	A full description of the statistical parameters including central tendency (e.g. means) or other basic estimates (e.g. regression coefficient) AND variation (e.g. standard deviation) or associated estimates of uncertainty (e.g. confidence intervals)
☐	☒	For null hypothesis testing, the test statistic (e.g. *F*, *t*, *r*) with confidence intervals, effect sizes, degrees of freedom and *P* value noted<br>*Give P values as exact values whenever suitable.*
☒	☐	For Bayesian analysis, information on the choice of priors and Markov chain Monte Carlo settings
☒	☐	For hierarchical and complex designs, identification of the appropriate level for tests and full reporting of outcomes
☒	☐	Estimates of effect sizes (e.g. Cohen's *d*, Pearson's *r*), indicating how they were calculated

Our web collection on statistics for biologists contains articles on many of the points above.

Software and code

Policy information about availability of computer code

Data collection	All electroencephalography & electromyograph recordings were done using OpenEx Software Suite (Tucker Davis Technologies, USA) as described and referenced in ''Sleep detection: data acquisition and online sleep state differentiation'' sections in Methods.
Data analysis	Statistical analyses were performed using Prism 6.01 (GraphPad Software, San Diego, CA, USA). All electroencephalography & electromyograph recordings were further edited for presentation using codes generated by MATLAB as described in ''Sleep detection: data acquisition and online sleep state differentiation'' sections in Methods.

For manuscripts utilizing custom algorithms or software that are central to the research but not yet described in published literature, software must be made available to editors and reviewers. We strongly encourage code deposition in a community repository (e.g. GitHub). See the Nature Portfolio guidelines for submitting code & software for further information.

Data

Policy information about availability of data

All manuscripts must include a data availability statement. This statement should provide the following information, where applicable:

- Accession codes, unique identifiers, or web links for publicly available datasets
- A description of any restrictions on data availability
- For clinical datasets or third party data, please ensure that the statement adheres to our policy

All data needed to evaluate the conclusions in the paper are present in the paper and the Supplementary Materials: Supplementary Data 1 and Supplementary Data 2. All resources and codes that supported the findings of this study are available from the corresponding author upon reasonable request.

Human research participants

Policy information about studies involving human research participants and Sex and Gender in Research.

Reporting on sex and gender	NA
Population characteristics	NA
Recruitment	NA
Ethics oversight	NA

Note that full information on the approval of the study protocol must also be provided in the manuscript.

Field-specific reporting

Please select the one below that is the best fit for your research. If you are not sure, read the appropriate sections before making your selection.

☒ Life sciences ☐ Behavioural & social sciences ☐ Ecological, evolutionary & environmental sciences

For a reference copy of the document with all sections, see nature.com/documents/nr-reporting-summary-flat.pdf

Life sciences study design

All studies must disclose on these points even when the disclosure is negative.

Sample size	No statistical method was used to pre-determine sample sizes. We determined sample sizes based on the previous literature. The number of animals in each experimental group are standard sample sizes in this field.
Data exclusions	Some animals were excluded from behavioral results if mice showed bias to either objects during training or from pharmacological results if the injection trace could not be seen in the post-hoc histological examination as explained in the ''Exploration analysis'' and ''Histology'' sections in Methods.
Replication	All the experiments were replicated in multiple runs for at least two times.
Randomization	Animals were randomly allocated into different experimental groups.
Blinding	The experimenter was not blinded in this study.

Reporting for specific materials, systems and methods

We require information from authors about some types of materials, experimental systems and methods used in many studies. Here, indicate whether each material, system or method listed is relevant to your study. If you are not sure if a list item applies to your research, read the appropriate section before selecting a response.

Materials & experimental systems

n/a	Involved in the study	
☐	☒	Antibodies
☒	☐	Eukaryotic cell lines
☒	☐	Palaeontology and archaeology
☐	☒	Animals and other organisms
☒	☐	Clinical data
☒	☐	Dual use research of concern

Methods

n/a	Involved in the study	
☒	☐	ChIP-seq
☒	☐	Flow cytometry
☒	☐	MRI-based neuroimaging

Antibodies

Antibodies used	- rabbit DsRed anti-mCherry (632496) - Takara Bio, Japan<br>- donkey anti-rabbit IgG Alexa Fluor 488 (A-21206) - Thermo Fisher Scientific, USA
Validation	*Describe the validation of each primary antibody for the species and application, noting any validation statements on the manufacturer's website, relevant citations, antibody profiles in online databases, or data provided in the manuscript.*

Animals and other research organisms

Policy information about studies involving animals; ARRIVE guidelines recommended for reporting animal research, and Sex and Gender in Research

Laboratory animals	c-fos::tTA/KA1::Cre, KA1::Cre, C57BL/6J male mice were bred in University of Toyama Animal Center. Mice were (12-20) weeks old.
Wild animals	The study did not involve wild animals.
Reporting on sex	The study was conducted on male mice.
Field-collected samples	The study did not include samples collected from the field.
Ethics oversight	The Animal Care and Use Committee of the University of Toyama.

Note that full information on the approval of the study protocol must also be provided in the manuscript.
